# Worsening Asthma Outcomes in Australian Adults: A Comparison of Stratified Sample Surveys in 2012 and 2021

**DOI:** 10.5694/mja2.70221

**Published:** 2026-06-23

**Authors:** Helen K. Reddel, Maria R. Ampon, Leanne M. Poulos, Sharon R. Davis, Brett G. Toelle, Guy B. Marks, Taehoon Lee

**Affiliations:** ^1^ Australian Centre for Airways Disease Monitoring (ACAM) Woolcock Institute of Medical Research Sydney New South Wales Australia; ^2^ Macquarie Medical School, Macquarie University Sydney New South Wales Australia; ^3^ Respiratory and Environmental Epidemiology Woolcock Institute of Medical Research Sydney New South Wales Australia; ^4^ Department of Health Sciences Macquarie University Sydney New South Wales Australia; ^5^ Lung Health Burnet Institute Melbourne Victoria Australia; ^6^ Division of Respiratory and Critical Care Medicine, Department of Internal Medicine Ulsan University Hospital, University of Ulsan College of Medicine, Ulsan Ulsan South Korea

**Keywords:** asthma, health status indicators, surveys and questionnaires, treatment outcome

## Abstract

**Objectives:**

To report patterns of asthma control, medications and healthcare utilisation in Australian adults with asthma in 2021, and assess changes since a similar survey in 2012.

**Study Type:**

Cross‐sectional web‐based survey (February–March 2021; *n* = 5427), compared with a similar 2012 survey (*n* = 2686).

**Setting/Participants:**

Adults (≥ 18 years) with asthma, recruited from large web‐based panels, with enrolment stratified by age group, gender and state/territory.

**Main Outcome Measures:**

Asthma control test (ACT), healthcare utilisation and medications.

**Results:**

Median age was 46 years; 59% of participants reported female gender. Compared with 2012, fewer participants had well‐controlled symptoms (ACT ≥ 20: 2021, 48.0%; 2012, 54.4%; *p* < 0.001), and more had very poorly controlled symptoms (ACT 5–15: 2021, 26.8%; 2012, 22.9%; *p* < 0.001). Urgent asthma healthcare had increased (2021, 37.9%; 2012, 28.6%; odds ratio 1.53 [95% confidence interval, 1.37–1.69]; *p* < 0.001). Inhaled corticosteroid (ICS) use in the previous year was similar (2021, 60.9%; 2012, 60.8%) but adherence was lower (*p* < 0.001). Fewer participants had good symptom control while taking little/no ICS (2021, 33.4%; 2012, 40.1%), and more had uncontrolled symptoms with little/no ICS (2021, 38.1%; 2012, 25.6%; *p* < 0.001); among the latter group, urgent healthcare utilisation had increased (2021, 63.5%; 2012, 41.2%; *p* < 0.001). In 2021, 28.7% reported using oral corticosteroids for asthma in the previous year; only 42.0% of ICS users recalled their inhaler technique having been checked in the past 12 months. Overuse of short‐acting beta_2_‐agonists was common: 56.3% adults obtained ≥ 3 inhalers in the previous year, and 10.5% obtained ≥ 12 inhalers. For symptom relief in the previous 4 weeks, only 13.3% adults reported using an anti‐inflammatory reliever (ICS–formoterol).

**Conclusion:**

Our comparison of these two large nationally stratified sample surveys demonstrates significant worsening of key asthma indicators between 2012 and 2021, including worse symptom control and urgent healthcare use, but also indicates opportunities for improvement. The findings highlight an urgent need for system‐wide implementation of the 2025 Australian asthma guidelines to reduce preventable morbidity.

**Trial Registration:**

ACTRN12620000977976p

## Plain Language Summary

**The known**: The first national survey of asthma control in Australian adults in 2012 found uncontrolled asthma symptoms in 46% of participants; 29% had required urgent medical care in the previous year, and only 34% were taking regular inhaled corticosteroids (ICS).

**The new**: A repeat cross‐sectional survey in 2021 found worse asthma control (uncontrolled symptoms 52%; urgent healthcare 37%). ICS adherence had decreased (29%). Overuse of short‐acting beta_2_‐agonists was common (56%), and only 13% of participants were using a best‐practice combination ICS–formoterol anti‐inflammatory reliever.

**The implications**: This comparison demonstrates an increasingly high burden of preventable morbidity from asthma in Australia. There are substantial opportunities for improvement with system‐wide implementation of recent major changes in Australian asthma guidelines published in 2025.

## 1 Introduction

Asthma is one of the most common chronic conditions in Australia, with a prevalence of 11% in adults and children—one of the highest globally—contributing to a substantial burden for people with asthma, their families and the healthcare system [1]. In 1989, following epidemics of asthma deaths in the 1960s–1980s [2], Australia published the first national asthma guidelines, recommending that all patients with persistent asthma symptoms (e.g., more than twice/week) should receive inhaled corticosteroids (ICS) [3]. Over the following 10 years, asthma deaths nearly halved. However, since 2006, progress has plateaued, apart from a temporary dip during the COVID‐19 pandemic [1].

Long‐term asthma control, comprising both recent symptom control and reduced exacerbation risk, is an important goal of therapy [4]. However, in the first national survey of asthma control in Australia in 2012 (2686 adults with asthma), we found that nearly half of the adults had uncontrolled asthma symptoms, almost 30% had required urgent medical care for asthma in the previous 12 months, and only one‐third were regularly using ICS [5]. Related commentary estimated that a million Australian adults had uncontrolled asthma and called for structural reform in asthma healthcare delivery. Suggested changes included re‐design of access to short‐acting beta_2_‐agonists (SABA) and ICS, promotion of combination ICS–formoterol in place of SABA and proactive clinical reviews for patients whose asthma remains uncontrolled despite taking ICS [6].

The 2012 findings also provided impetus for the National Asthma Council and Asthma Australia to jointly develop the *National Asthma Strategy 2018* [7], calling for action to support effective self‐management practices, develop the health professional workforce, enhance asthma care, create supportive community environments and promote research [7]. Progress would be assessed against 10 National Asthma Indicators [8], including routinely recorded administrative data such as hospitalisations, emergency department (ED) presentations, deaths, medication dispensing and costs of asthma, as well as asthma control and quality of life [8].

In light of these calls for action, we aimed to investigate patterns of asthma control, medication use and healthcare utilisation in a large demographically stratified sample of Australian adults with asthma. Specifically, we aimed to assess whether asthma control in Australian adults had changed since 2012 and identify potential areas for improvement.

## 2 Methods

### 2.1 Study Design and Inclusion Criteria

We conducted a cross‐sectional web‐based survey in February/March 2021, recruiting participants from large web‐based panels (Dynata, Melbourne, ~1,600,000 Australian members) [9], with reporting consistent with recommendations for web‐based surveys (see the Checklist for Reporting Results of Internet E‐Surveys [CHERRIES] in Table S1 [10]). The methods for the 2021 survey were similar to those used in our 2012 cross‐sectional survey (Table S1) [5]. To avoid selection bias, panel members were emailed a request to ‘complete a survey’, without further details. Respondents were asked if they had any of five conditions, including asthma. Current asthma was defined, as in the Australian Bureau of Statistics National Health Surveys (NHS), by positive responses to (a) ‘Have you ever been told by a doctor or a nurse that you have asthma?’ and (b) ‘Have you had symptoms of asthma or taken medication for asthma in the last 12 months?’ Sampling was stratified by age group, gender and state/territory of residence to obtain a nationally stratified sample of adults with asthma (aged ≥ 18 years) based on asthma‐specific data from NHS 2017–18 [11].

### 2.2 Questionnaire

The survey instrument included existing tools and purpose‐designed questions and was piloted in a 10% sample. Asthma symptom control was assessed with the Asthma Control Test (ACT) [12]. Asthma‐related healthcare utilisation in the previous 12 months included non‐urgent general practitioner (GP) visits and urgent healthcare (urgent GP visits and hospital or ED visits). Participants were shown colour images and brand names of all respiratory medications available in Australia at the time, and asked, ‘Which of these treatments have you used for asthma in the past 12 months?’ They were asked about medication(s) used for symptom relief, the number of SABA inhalers obtained in the past year and, for ICS‐containing medications, the frequency of use in the previous 4 weeks, and barriers to their use.

### 2.3 Data Analysis

Data were analysed using SAS version 9.4 (SAS Institute, Cary, NC). The study population was weighted to the population of Australian adults with asthma from NHS surveys (2011 and 2017–2018) to obtain prevalence estimates by age group, gender and state/territory of residence [11]. Data for eight respondents were excluded because they selected ‘other’ for the question about gender; this option was not offered in the 2012 survey. Statistical comparisons between 2021 and 2012 were conducted using individual patient data [5], using chi‐squared tests and univariate logistic regression models to calculate odds ratios, with adjustment for age group, gender and the socio‐economic indices for area (SEIFA) Index of Relative Socio‐economic Disadvantage quintile based on postcode of residence. For comparison with 2012 data, participants were classified into four groups based on symptom control (ACT ≥ 20 or < 20) and use of ICS (≥ 5 or 0–4 days/week). The proportion in each group that had used urgent healthcare in the previous 12 months was compared with logistic regression analyses as above. *p* values of < 0.05 were considered statistically significant.

Ethics approval was obtained from the Human Research Ethics Committee, University of Sydney, NSW (2020‐630). All participants provided informed consent.

Some data from the 2021 survey have been previously reported in an analysis specifically comparing data for participants who satisfied criteria for difficult‐to‐treat asthma and those who did not [9]. For that analysis, the study population was weighted by SEIFA in addition to age group, gender and state of residence (weighted total, 6048 participants) (Figure S1).

## 3 Results

A total of 5427 adults with current asthma, with a median (interquartile range) age of 46 (33–62) years participated in the 2021 survey (Figure S1, Table S1). Demographic data closely matched NHS data for adults with asthma (Table S2) and were similar to the 2012 survey, apart from the expected increase in age (Table 1).

**TABLE 1:** Demographics, asthma control and quality of life indicators, 2012 and 2021.

	2012	2021	*p* *
Number of participants	*N* = 2686	*N* = 5427	
Age group, years, *n* (%)			< 0.001
< 20^a^	207 (7.7)	116 (2.1)	
20–29	493 (18.4)	848 (15.6)	
30–39	523 (19.5)	985 (18.1)	
40–49	377 (14.0)	1056 (19.5)	
50–59	441 (16.4)	848 (15.6)	
60–69	344 (12.8)	979 (18.0)	
70+	302 (11.2)	596 (11.0)	
Self‐reported gender,^b^ *n* (%)			0.08
Female	1534 (57.1)	3209 (59.1)	
Male	1152 (42.9)	2218 (40.9)	
Government concession card, *n* (%)	1473 (54.8)	3001 (55.3)	0.58
Asthma control test (ACT)			
ACT score, mean ± SD	19.19 ± 4.62	18.46 ± 4.58	< 0.001
Asthma symptom control, *n* (%)			< 0.001
Very poorly controlled (ACT 5–15)	615 (22.9)	1456 (26.8)	
Partly controlled (ACT 16–19)	609 (22.7)	1367 (25.2)	
Well controlled (ACT 20–25)	1461 (54.4)	2604 (48.0)	
Interference with activity^c^			< 0.001
All of the time	46 (1.7%)	277 (5.1%)	
Most of the time	164 (6.1%)	358 (6.6%)	
Some of the time	340 (12.7%)	879 (16.2%)	
A little of the time	559 (20.8%)	1494 (27.5%)	
None of the time	1577 (58.7%)	2419 (44.6%)	
General health status, *n* (%)			< 0.001
Excellent	218 (8.1)	637 (11.7)	
Very good	872 (32.5)	1642 (30.2)	
Good	1050 (39.1)	1906 (35.1)	
Fair	402 (15.0)	1001 (18.4)	
Poor	145 (5.4)	242 (4.5)	

*Note:* Any differences between sum of numbers and total number of participants are due to rounding of weighted data.

Abbreviation: SD, standard deviation.

^a^ In 2012, 16–19 years; in 2021, 18–19 years.

^b^ In the 2012 survey, the options for gender were ‘Male’ and ‘Female’. In 2021, the options were ‘Male’, ‘Female’ and ‘Other (please specify)’. Eight participants selected ‘Other’. Data for these participants were not able to be included in other analyses due to lack of national data for people with asthma who do not identify as male or female.

^c^ From Asthma Control Test Q1: ‘In the past 4 weeks, how much of the time did your asthma keep you from getting as much done at work, school or at home?’

* Chi‐squared analysis for all variables except for Asthma Control Test score. For this, a generalised linear model was used to test difference between ACT scores for 2012 and 2021, adjusted for age‐group, gender and Socio‐Economic Indices for Areas (SEIFA) Index of Relative Socio‐economic Disadvantage.

Asthma symptom control was worse in 2021, indicated by lower ACT scores, with 26.8% (1456/5427) of participants having very poorly controlled symptoms (ACT 5–15) compared with 22.9% (615/2686) in 2012. Fewer than half of participants in 2021 had well‐controlled asthma symptoms (Table 1). Quality of life was worse in the 2021 survey, with more participants reporting that asthma interfered with daily activity some/most/all the time (27.9%, 1513/5427) compared with 20.5% (550/2686). Similarly, fair or poor health status was reported by 22.9% (1243/5427) of participants in 2021 versus 20.3% (546/2686) in 2012 (Table 1).

**FIGURE 1:**
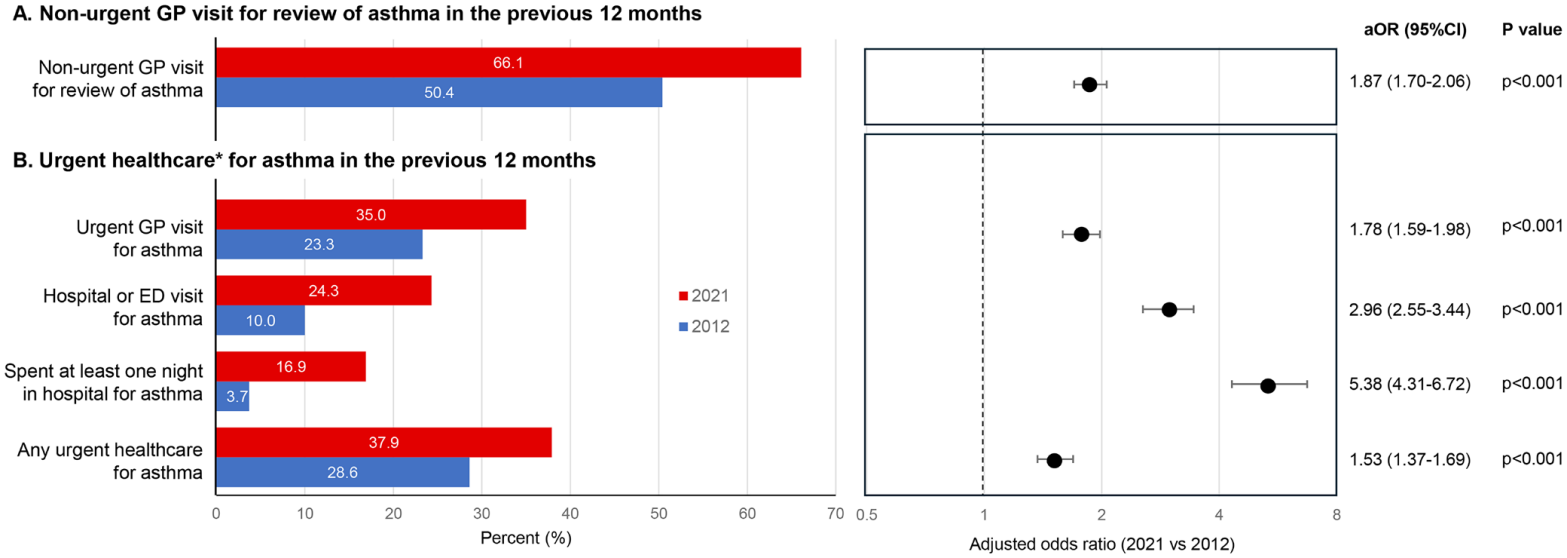
Healthcare for asthma in the previous 12 months. *Note that self‐reported data for ‘spent at least one night in hospital for asthma’ includes overnight emergency department stays as well as formal hospital admissions. aOR, adjusted odds ratio; CI, confidence interval; ED, emergency department; GP, general practitioner. Logistic regression analyses were adjusted for age‐group, gender and Socio‐Economic Indexes for Areas (SEIFA) Index of Relative Socio‐economic Disadvantage quintile. Data are in Table S3.

The proportion of participants reporting a non‐urgent GP review for asthma in the previous year increased to about two‐thirds in 2021 from half in 2012 (Figure 1; Table S3). However, urgent healthcare utilisation for asthma rose substantially. In 2021, 37.9% of participants (2056/5427) reported at least one urgent asthma‐related visit to the GP, ED or hospital, compared with 28.6% (769/2686) in 2012 (odds ratio [OR] 1.53, 95% confidence interval [CI] 1.37–1.69, *p* < 0.001) (Figure 1, Table S3).

Use of any ICS in the previous year was reported by 60.9% (3305/5427) of participants, unchanged from 60.8% (1634/2686) in 2012. However, adherence was worse, with fewer participants taking ICS ≥ 5 days/week (Table 2).

**TABLE 2:** Asthma medications, 2012 and 2021.

Asthma medications used in past 12 months, *n* (%)	2012	2021	*p* *
Number of participants	*N* = 2686	*N* = 5427	
ICS‐containing medications in the last 12 months			
Any ICS‐containing medication	1634 (60.8)	3305 (60.9)	0.21
ICS‐only inhaler	448 (16.7)	874 (16.1)	0.75
ICS‐LABA (combination inhaler)	1332 (49.9)	2713 (50.0)	0.26
ICS + LABA (separate inhalers)	39 (1.5)	198 (3.7)	< 0.001
ICS + LAMA (separate inhalers)	38 (1.4)	116 (2.1)	0.20
ICS + LABA + LAMA (separate or combination)	175 (6.5)	369 (6.8)	0.81
In the last 12 months, health professional has checked ICS inhaler being used correctly	N/A	1387/3305 (42.0)	—
Long‐acting bronchodilator(s) with no ICS in the last 12 months	50 (1.8)	304 (5.6)	< 0.001
LABA‐only	15 (0.6)	159 (2.9)	< 0.001
LAMA‐only	33 (1.2)	83 (1.5)	0.62
LABA‐LAMA	2 (0.1)	212 (3.9)	< 0.001
LTRA with no ICS	6 (0.2)	16 (0.3)	0.76
Theophylline with no ICS	11 (0.4)	23 (0.4)	0.82
OCS with no ICS	4 (0.1)	99 (1.8)	< 0.001
OCS course (≥ 3 days) for asthma in the last 12 months			
≥ 1 course	N/A	1555 (28.7%)	—
≥ 3 courses	N/A	451 (8.3%)	—
Any SABA used in the last 12 months	2475 (92.2)	4508 (83.1)	< 0.001
Answered question about SABA purchase in the previous 12 months (2021)	N/A	3987/4508 (88.4)	—
Obtained ≥ 3 SABA inhalers in previous 12 months [% of all adults with asthma, *n* = 5427]	N/A	2246/3987 (56.3) [41.4]	—
Obtained ≥ 12 SABA inhalers in previous 12 months [% of all adults with asthma, *n* = 5427]	N/A	420/3987 (10.5) [7.7]	—
Medication(s) used for symptom relief in the past 4 weeks^a^		*N* = 3679/5427 (67.8%)	
SABA	N/A	2970/3679 (80.7)	—
ICS–formoterol	N/A	489/3679 (13.3)	—
ICS–formoterol with no SABA	N/A	231/3679 (6.3%)	—
Other medications, with no SABA or ICS–formoterol^b^	N/A	478/3679 (13.0)	—
ICS‐containing medication used ≥ 5 days/week^c^	911/2686 (33.9%)	1555/5427 (28.6%)	< 0.001

Abbreviations: ICS, inhaled corticosteroid; LABA, long‐acting beta_2_‐agonist; LAMA, long‐acting muscarinic antagonist; LTRA, leukotriene receptor antagonist (montelukast); N/A, not available in 2012; OCS, oral corticosteroids; SABA, short‐acting beta_2_‐agonist, e.g., salbutamol.

^a^ More than one medication could be selected in response to this question. 258/489 (52.7%) of participants who reported using ICS–formoterol for symptom relief in the previous 4 weeks also reported using SABA during that time; hence, only 231/3679 (6.3%) of participants who used any medication for symptom relief were using an anti‐inflammatory reliever according to guidelines (i.e., instead of SABA).

^b^ Other medications that 478 (13.0%) participants reported using for relief of asthma symptoms and who did not also use SABA or ICS–formoterol, included ipratropium, ICS (alone), non‐formoterol ICS–LABA combinations, formoterol, other long‐acting bronchodilators (LABA and/or LAMA), OCS, theophylline, montelukast, antihistamines, cough/cold/flu preparations, analgesics and antibiotics.

^c^ In 2021, participants were asked about frequency of medication use for each ICS‐containing medication they had used in the previous 4 weeks; in 2012, no timeframe was specified (‘How often do you take your [medication name]?’).

* *p* values were calculated from logistic regression adjusting for design (2021 vs. 2012), age group, gender and Socio‐Economic Indices for Area (SEIFA) Index of Relative Socio‐economic Disadvantage.

**FIGURE 2:**
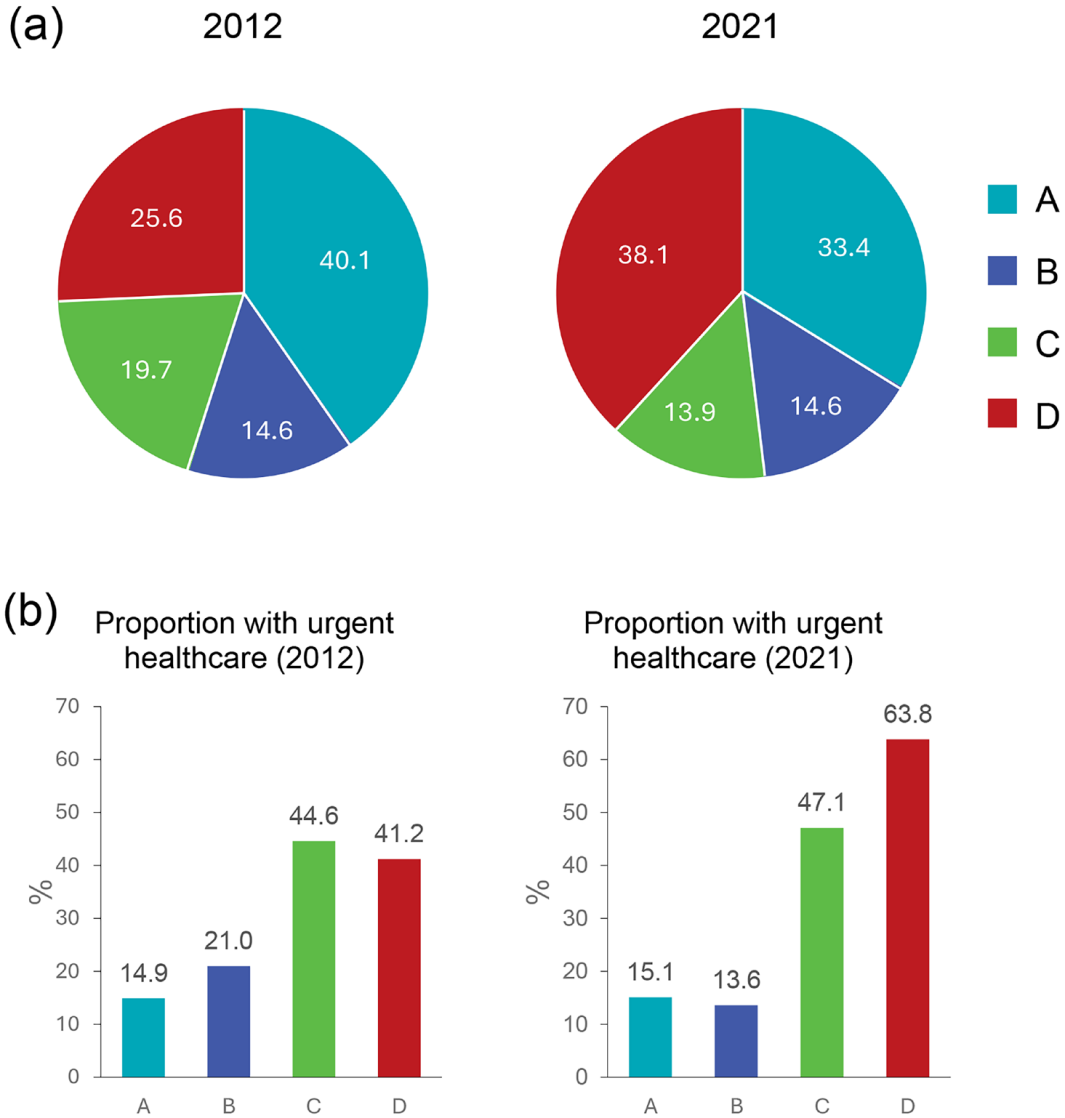
Four patient groups in 2012 and 2021, (a) classified by symptom control and preventer use, and (b) showing the proportion of each group requiring urgent healthcare for asthma. For comparison with survey results in 2012, participants were classified by asthma symptom control (Asthma Control Test [ACT] ≥ 20 and < 20) and self‐reported adherence with ICS (≥ 5 and 0–4 days/week). Group A: Good symptom control with no ICS or poor ICS adherence. Group B: Good symptom control with good self‐reported ICS adherence. Group C: Poor symptom control despite good self‐reported ICS adherence. Group D: Poor symptom control with no ICS or poor ICS adherence. (a) The difference in distribution of groups A–D between 2012 and 2021 (*p* < 0.001) was analysed by a logistic regression model, testing for the interaction between year and group, adjusted for age group, gender and Socio‐Economic Indices for Area (SEIFA) Index of Relative Socio‐economic Disadvantage. (b) Within each group, the difference in risk of urgent healthcare between 2012 and 2021 was analysed by a logistic regression model, adjusted for age group, gender and SEIFA. Group A: *p* = 0.155; Group B: *p* = 0.031; Group C: *p* = 0.672; Group D: *p* < 0.001. Additional data for these figures are in Table S5.

When participants were classified based on symptom control (ACT ≥ 20 and < 20) and ICS use (≥ 5 and 0–4 days/week) (Figure 2, Table S5), fewer participants had good symptom control while taking no/little ICS‐containing therapy (Group A, ‘mild’ asthma) in 2021, compared with 2012. The proportions of participants with well‐controlled symptoms and good adherence (Group B) were similar in 2021 and 2012, and the proportion with uncontrolled symptoms (ACT score < 20) despite good ICS adherence (Group C) was lower. However, there was a substantial increase in the proportion of participants with uncontrolled symptoms who were using little/no ICS (Group D), comprising almost 40% in 2021 (2021 vs. 2012, Groups A–D, overall *p* < 0.001). All groups reported urgent healthcare for asthma, including almost two‐thirds of Group D, significantly more than in 2012 (*p* < 0.001). Notably, 14%–15% of participants with good recent symptom control also reported urgent healthcare in the previous year (Figure 2B, Table S5C).

### 3.1 Additional Data About Medications (2021)

In 2021, two‐thirds of participants (3679/5427, 67.8%) reported using any medication for asthma symptom relief in the previous 4 weeks. The medication used to relieve symptoms was SABA for 80.7% (2970/3679). A combination ICS–formoterol reliever was reported by only 13.3% (489/3679); over half had also used SABA (Table 2).

Overuse of SABA was widespread: 56.3% (2246/3987) of SABA users reported obtaining ≥ 3 SABA inhalers in the past 12 months, and 10.5% (420/3987) obtained ≥ 12 inhalers (Table 2). Urgent healthcare for asthma increased with SABA overuse, being reported by 43.3% (973/2246) and 52.1% (219/420) of those obtaining ≥ 3 and ≥ 12 SABA inhalers, respectively, compared with 34.0% (1083/3181) among those obtaining 0–2 SABA inhalers. The odds ratios for urgent healthcare were 1.54 (CI, 1.37–1.73) with ≥ 3 SABA inhalers and 2.46 (CI, 1.94–3.11) with ≥ 12 inhalers.

Overall, 28.7% (1555/5427) of surveyed adults reported having taken ≥ 1 course of oral corticosteroids (OCS) for asthma in the previous 12 months; 8.3% (451/5427) took ≥ 3 courses (Table 2).

Among ICS users, the most common reasons reported for not taking it as prescribed were feeling well in the moment, not thinking they needed it, forgetting to take it, or cost (Table S4). Only 42.0% (1387/3305) of ICS users reported that, in the past year, a healthcare professional had checked that they were using their inhaler correctly.

As previously reported [9], participants from the 2021 survey who had guideline‐defined difficult‐to‐treat asthma had a much higher burden of symptoms, exacerbations, healthcare utilisation and OCS exposure compared with participants who did not have difficult‐to‐treat asthma.

## 4 Discussion

This survey of 5427 Australian adults with asthma, nationally stratified by age group, gender and state/territory of residence, provides concerning evidence of worse asthma‐related outcomes in 2021 compared with a similar survey we conducted in 2012 [5]. Asthma symptom control had declined, with fewer than half of the adults now having well‐controlled symptoms. Quality of life was worse in 2021, with asthma interfering more with day‐to‐day activities and impacting more on health status. Only 60% reported taking any ICS‐containing medication in the previous 12 months, and adherence was lower, with fewer than 30% taking ICS most/all days in the previous 4 weeks. Despite a reported increase in non‐urgent GP visits, urgent healthcare for asthma had increased by 53% (from 28.6% to 37.9%, adjusted OR, 1.53 [CI, 1.37–1.69], Figure 1). This was particularly common among patients with poor symptom control who were taking little/no ICS, but urgent care was also needed by some patients with good symptom control. New data in 2021 found that SABA overuse was common, with 56% of adult SABA users obtaining ≥ 3 inhalers in the previous 12 months. Only 13% participants reported use of an anti‐inflammatory reliever (combination ICS–formoterol), which is the preferred treatment strategy in the new 2025 Australian asthma guidelines [13, 14]. The findings raise serious concerns about asthma management in Australia, but highlight major opportunities for improving outcomes if the new guidelines are widely implemented.

The significant deterioration of patient‐reported asthma outcomes in Australia between 2012 and 2021 contrasts with shorter‐term trends on the Australian Institute of Health and Welfare (AIHW) asthma dashboard. ‘Progress’ is reported on asthma‐related hospitalisations, ED visits and asthma‐related deaths between 2016/17 and 2021/22 [15]. This may reflect short‐term reductions in respiratory mortality, hospitalisations and ED visits seen in many countries during the COVID‐19 pandemic [16]. Progress is also reported by AIHW on sub‐optimal asthma control (from Pharmaceutical Benefits Scheme [PBS] prescribing of ≥ 3 reliever inhalers in 12 months to people aged ≤ 40 years, i.e., SABA overuse, which decreased from 29% to 27%); and on quality of life from interference with daily activities ≥ 2 times in the past 4 weeks [15]. By contrast, from patient‐reported survey responses in 2021 versus 2012, we found a significant worsening of asthma symptom control based on an internationally accepted criterion (ACT < 20) [4], increased interference by asthma with activity some/most/all the time, and ~50% increase in reported urgent healthcare in the last year.

There are limited comparable data on trends in asthma outcomes from other countries. In New Zealand, severe exacerbations (primary hospital discharge diagnosis of asthma or OCS prescription) increased over 2010–2019 [17]. From UK primary care, exacerbations increased between 2006 and 2016 [18], and OCS prescribing for asthma increased between 2005 and 2019 [19]. We found no longitudinal data on ACT scores, but a cohort study found that variability in ACT score over 3 years was associated with worse outcomes [20].

No obvious explanation emerged from the 2021 survey for the worsening in asthma outcomes, except for a further reduction in already‐low adherence. There has been increasing pressure on GPs due to increased prevalence of chronic disease, workforce shortages and gaps in funding and infrastructure, and more patients are delaying care for chronic conditions [21]. However, more participants reported non‐urgent GP visits for asthma than in 2012, indicating the opportunity for intervention. Although the proportion of participants using SABA at any time in the previous 12 months was lower in 2021 than in 2012, our survey confirmed that SABA overuse in Australia [22] is among the highest globally: 56% of SABA users reported obtaining ≥ 3 SABA inhalers in the previous year (nearly double the Australian Institute of Health and Welfare [AIHW] PBS‐based estimate), and 10% of SABA users reported obtaining ≥ 12 canisters. SABA overuse is associated with increased exacerbations [22, 23, 24] (confirmed from our 2021 survey). From international surveys, SABA overuse is associated with hospitalisations and asthma mortality [22, 23]; a Swedish study reported that use of ≥ 11 canisters was associated with a hazard ratio of 31.72 for asthma‐related death [23]. Together, these data suggest that reliance on and overuse of SABA is a major contributor to poor asthma outcomes in Australia. Contributing factors include its low cost, over‐the‐counter availability and the ability to obtain 12 SABA inhalers with a single, unrestricted PBS prescription. Few clinicians or patients are aware that regular SABA use, even for 1–2 weeks, increases airway hyperresponsiveness and reduces bronchodilator effect [25], which can set up a vicious cycle encouraging SABA overuse.

Management of any chronic condition in general practice would benefit from improved models of care. The National Asthma Strategy 2018 called for comprehensive changes to support effective self‐management practices; develop the health professional workforce; create an integrated, equitable and accessible healthcare system; provide supportive community environments; and increase support for research, evidence and data [7]. More recently, key stakeholder groups have called for innovations such as digital tools for improved GP assessment and management, greater patient involvement and advocacy, use of electronic and social media platforms for asthma education and increased involvement of pharmacists [26, 27]. Current liberal access to SABA by prescription and over‐the‐counter could (and should) be restricted on safety grounds, but this would be strongly resisted by patients (as pharmacists experienced during the SABA restrictions in the COVID‐19 pandemic) if they were not aware of, and confident in, an alternative asthma reliever. Trust in SABA is not surprising—it is usually the first asthma medication patients experience, and qualitative research consistently finds that the rapidity of its symptom relief establishes patients' belief that it is their main asthma treatment [28, 29, 30]. In this context, poor adherence with ICS, if prescribed, is unsurprising, particularly given the higher cost and, with past guidelines, the requirement for daily use even when asymptomatic [29, 30].

Taken together, a paradigm shift in asthma management is required to overcome the entrenched beliefs, behaviours and health system practice regarding SABA and ICS in Australia. Such a paradigm change occurred in 2019 when international asthma guidelines recommended against SABA‐only treatment, and recommended the anti‐inflammatory reliever (AIR) strategy with ICS–formoterol instead of SABA‐based therapy [31, 32]. This evidence‐based approach is now preferred by many guidelines (e.g., New Zealand 2020 [33], United Kingdom 2024 [34], China 2024 [35]) and most recently Australia (September 2025) [13, 14].

**FIGURE 3:**
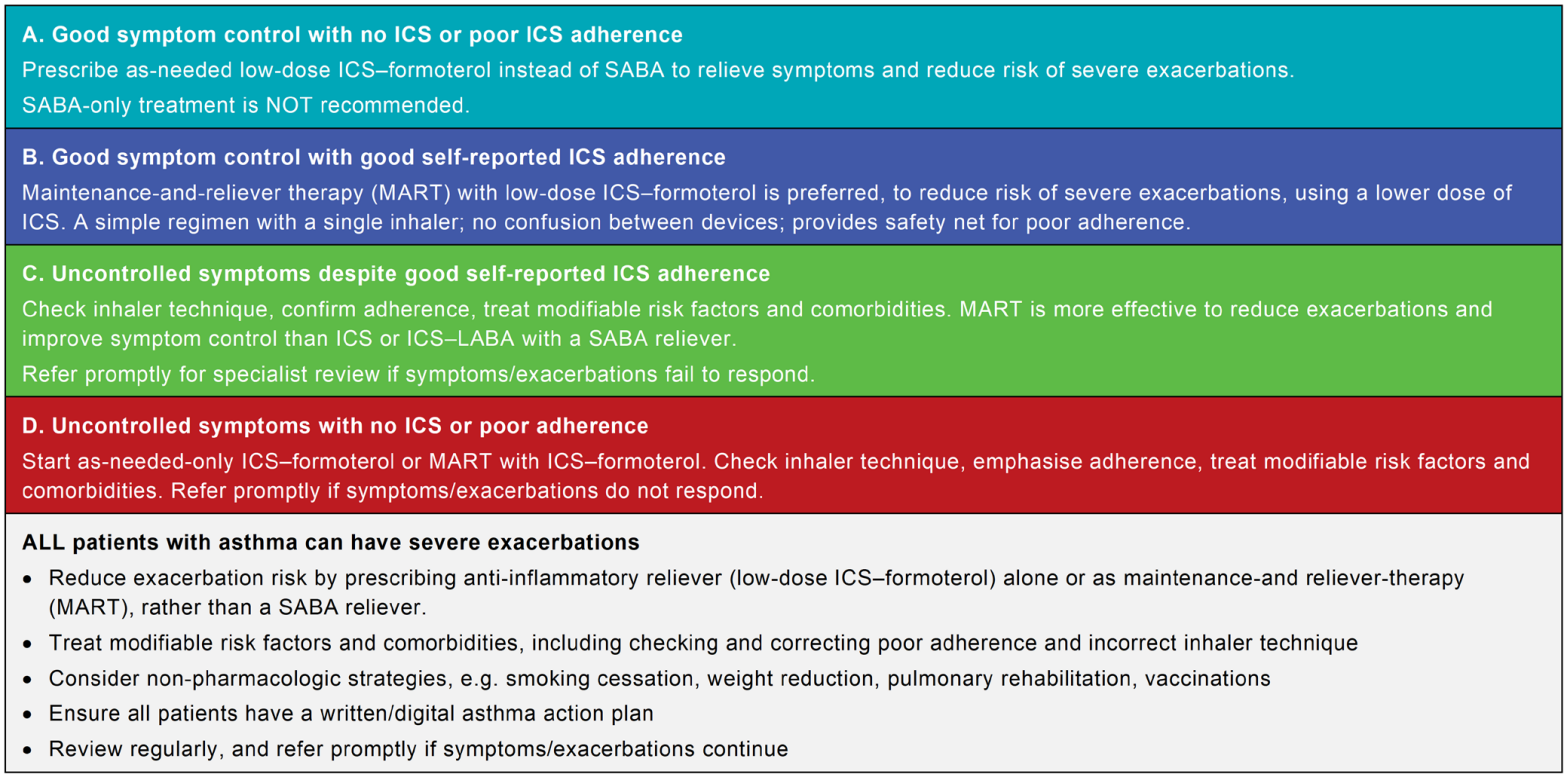
Current clinical recommendations for adults with asthma in Groups A–D, Figure 2 ^a^. Letters A–D and colours correspond to the segments in Figure 2. ICS, inhaled corticosteroid‐containing therapy (alone or in combination with long‐acting beta_2_‐agonist, LABA); MART, maintenance‐and‐reliever therapy with ICS–formoterol; SABA, short‐acting beta_2_‐agonist, e.g., salbutamol. ^a^Based on the Australian Asthma Handbook 2025 [12]. The present survey included only adults, but the above clinical recommendations apply also to adolescents.

The fundamental change that the new Australian guidelines represent is illustrated by the major changes in clinical recommendations for Groups A–D in Figure 3 [5]. For participants with good symptom control and little/no ICS (Group A), Australian guidelines now recommend ‘anti‐inflammatory treatment from day one’ [13] with as‐needed‐only low‐dose ICS–formoterol [14]. This has been found to reduce the risk of severe exacerbations and asthma‐related ED visits/hospitalisations by 65% compared with SABA‐only, and reduce the risk of ED visits/hospitalisations by 37% compared with daily ICS plus as‐needed SABA [36]. For Group B (good symptom control, good adherence), maintenance‐and‐reliever‐therapy with ICS–formoterol (MART) allows a lower ICS dose. For patients with uncontrolled symptoms despite good adherence (Group C), with nearly half requiring urgent healthcare, a switch to MART is recommended to reduce their high exacerbation risk [37], plus checking inhaler technique, adherence, modifiable risk factors and comorbidities [14]. Finally, for patients with uncontrolled symptoms and little/no ICS use (Group D), MART is the treatment of choice to reduce their very high exacerbation risk and OCS exposure. The AIR/MART strategy is forgiving of poor adherence, by ensuring that patients always receive ICS when they take their reliever, and it simplifies treatment, requiring only one inhaler device/dose. For Groups A–D, guidelines also recommend personalised treatment by identifying and managing treatable traits, and considering patient preferences and goals, with early specialist referral if symptoms or exacerbations do not respond promptly [14, 32]. The potential magnitude of benefit from implementing the new Australian guidelines, especially in patients currently using little/no ICS or over‐using SABA, is large.

However, uptake of the AIR approach in Australia is very low, despite PBS funding since 2007 for MART and June 2020 for AIR‐only. In our 2021 survey, only 13% of surveyed adults reported using any ICS–formoterol reliever; with half of these participants having also used SABA. Widespread dissemination, explanation and implementation of the new evidence‐based guidelines will be essential to improve asthma outcomes. International models provide some encouraging precedents. In New Zealand, dispensing of ICS–formoterol has increased substantially, and exacerbations are decreasing [38]. A UK primary care practice reported that asthma hospitalisations had decreased by 91.3% and OCS‐treated exacerbations by 82.1% among patients who switched to MART compared with those continuing with ICS–long‐acting beta_2_‐agonist (LABA) plus SABA reliever [39]. The co‐designed SENTINEL program in Hull, United Kingdom, prioritising asthma review visits for SABA overusers and switching to MART, has substantially reduced SABA over‐prescribing, with prescribing of ≥ 3 inhalers/year dropping from 83.6% to 26.5% of patients, largely due to an increase in MART prescribing from 4.7% to 44.7%; early data show a reduction in exacerbations [40]. This program, expanded to include as‐needed‐only ICS–formoterol for mild asthma, is being implemented widely in the United Kingdom, and the experience could provide a model for change in Australia. Further system‐wide changes, including to minimise the cost differential between ICS–formoterol and SABA, decrease overuse of SABA in EDs and improve prescribing resources, will be needed to improve outcomes in Australia.

The strengths of our study include its large sample size, rigorous methodology to minimise selection bias, stratification by age group, gender and state/territory of residence and use of validated measures aligned with National Asthma Indicators. Patient‐reported data provide essential insights into asthma control and risk factors that administrative data cannot fully capture. Use of many of the same outcomes as in our 2012 survey [5] enabled reliable comparisons over time.

Limitations include the inherent constraints of survey research, such as exclusion of non‐English speakers, potential bias within quotas and reliance on self‐reported diagnosis and medications. Self‐reported adherence may be over‐estimated; if so, actual ICS usage may be even lower than reported. The 2021 survey was conducted during the COVID‐19 pandemic, which was associated with fewer asthma‐related hospital presentations. Therefore, the increase in urgent healthcare use since 2012 may be an underestimate.

## 5 Conclusions

This cross‐sectional survey of a large sample of Australian adults with asthma, stratified by age group, gender and state/territory of residence, revealed substantial worsening of key asthma indicators between 2012 and 2021, including poor symptom control and urgent healthcare utilisation. It also indicates opportunities for improvement, if the recent major changes in Australian asthma guidelines for adolescents and adults are widely implemented. For implementation to succeed and asthma outcomes to improve, system‐level issues must also be addressed urgently, particularly the current disparity in accessibility and cost between SABA inhalers and the safer and more effective combination ICS–formoterol options.

## Author Contributions

**Helen K. Reddel:** conceptualisation, formal analysis, funding acquisition, investigation, methodology, project administration, resources, supervision, validation, visualisation, writing – original draft, writing – review and editing. **Maria R. Ampon:** data curation, formal analysis, methodology, software, validation, writing – original draft, writing – review and editing. **Leanne M. Poulos:** methodology, project administration, resources, visualisation, writing – original draft, writing – review and editing. **Sharon R. Davis:** investigation, methodology, project administration, resources, writing – review and editing. **Brett G. Toelle:** methodology, writing – review and editing. **Guy B. Marks:** formal analysis, methodology, software, writing – review and editing. **Taehoon Lee:** conceptualisation, formal analysis, methodology, software, writing – original draft, writing – review and editing.

## Funding

The Australian Centre for Airways disease Monitoring (ACAM) is a unit of the Woolcock Institute of Medical Research, an independent medical research institute affiliated with Macquarie University. Support for the present research was provided via funding paid to the Woolcock Institute from the National Health and Medical Research Council (NHMRC) Centre for Research Excellence in Severe Asthma, and from investigator‐initiated research grants from AstraZeneca, GlaxoSmithKline, Chiesi and Sanofi. Helen Reddel is funded by a NHMRC Investigator Fellowship. The funding sources were not involved in the study design, data collection, analysis or interpretation, reporting or publication of this work.

## Disclosure

Not commissioned, externally peer reviewed.

## Consent

The patients provided written consent for publication.

## Conflicts of Interest

The Woolcock Institute of Medical Research has received independent investigator‐sponsored research grants from GlaxoSmithKline, AstraZeneca, Sanofi and Chiesi and funding from the National Health and Medical Research Council. In addition, Taehoon Lee's institution (Ulsan University Hospital) received Korean government (MIST) research funding from the National Research Foundation of Korea. Helen K. Reddel received honoraria for participation on advisory boards for AstraZeneca, GSK, Chiesi, Sanofi and Novartis and steering committees for AstraZeneca; for medical education from AstraZeneca, GSK, Getz, Cipla, Chiesi; and received drugs for an investigator‐sponsored study from AstraZeneca. She is Chair of the GINA Science Committee. Guy B. Marks is the President and Board Member for the International Union Against Tuberculosis and Lung Disease and has participated in advisory boards for AstraZeneca.

## Supporting information


**Figure S1:** Flow chart of study participants from February to March 2021 survey.
**Table S1:** CHERRIES checklist for 2012 and 2021 surveys.
**Table S2:** Demographics of populations included in 2012 and 2021 surveys compared with contemporaneous Australian government National Health Survey data for people with asthma.
**Table S3:** Healthcare utilisation for asthma in the previous 12 months, 2012 and 2021 (data for Figure 1).
**Table S4:** Reasons for poor adherence with ICS‐containing medications.
**Table S5:** Asthma symptom control and frequency of ICS‐containing preventer use in (A) 2012 and (B) 2021. (A) Asthma symptom control and frequency of ICS‐containing preventer use over the past 12 months in 2012 (*n* = 2654). (B) Asthma symptom control and frequency of ICS‐containing preventer use in 2021 (*n* = 5427). (C) Proportion of participants requiring urgent healthcare in the previous 12 months, by symptom control/adherence group in Figure 2b.

## Data Availability

The data that support this study's findings are available on reasonable request from the corresponding author. They are not publicly available due to ethical or privacy issues.
