# Supplementary material for: Worsening Asthma Outcomes in Australian Adults: A Comparison of Stratified Sample Surveys in 2012 and 2021
Source: Med J Aust. 2026 Jun 23;224(6):e70221. doi: 10.5694/mja2.70221 (PMC13291629; doi:10.5694/mja2.70221)
Supplement: Supplementary file 1 — Figure S1: Flow chart of study participants from February to March 2021 survey. Table S1: CHERRIES checklist for 2012 and 2021 surveys. Table S2: Demographics of populations included in 2012 and 2021 surveys compared with contemporaneous Australian government National Health Survey data for people with asthma. Table S3: Healthcare utilisation for asthma in the previous 12 months, 2012 and 2021 (data for Figure 1). Table S4: Reasons for poor adherence with ICS‐containing medications. Table S5: Asthma symptom control and frequency of ICS‐containing preventer use in (A) 2012 and (B) 2021. (A) Asthma symptom control and frequency of ICS‐containing preventer use over the past 12 months in 2012 (n = 2654). (B) Asthma symptom control and frequency of ICS‐containing preventer use in 2021 (n = 5427). (C) Proportion of participants requiring urgent healthcare in the previous 12 months, by symptom control/adherence group in Figure 2b. [file MJA2-224-0-s001.pdf]

Supporting Information

Supplementary material

This appendix was part of the submitted manuscript and has been peer reviewed. It is posted as supplied by the authors.

Appendix to: Reddel HK, Ampon MR, Poulos LM, et al. Worsening asthma outcomes in Australian adults: a comparison of stratified sample surveys in 2012 and 2021. *Med J Aust* 2026; doi: 10.5694/mja2.70221.

Figure S1: Flow chart of study participants from February/March 2021 survey

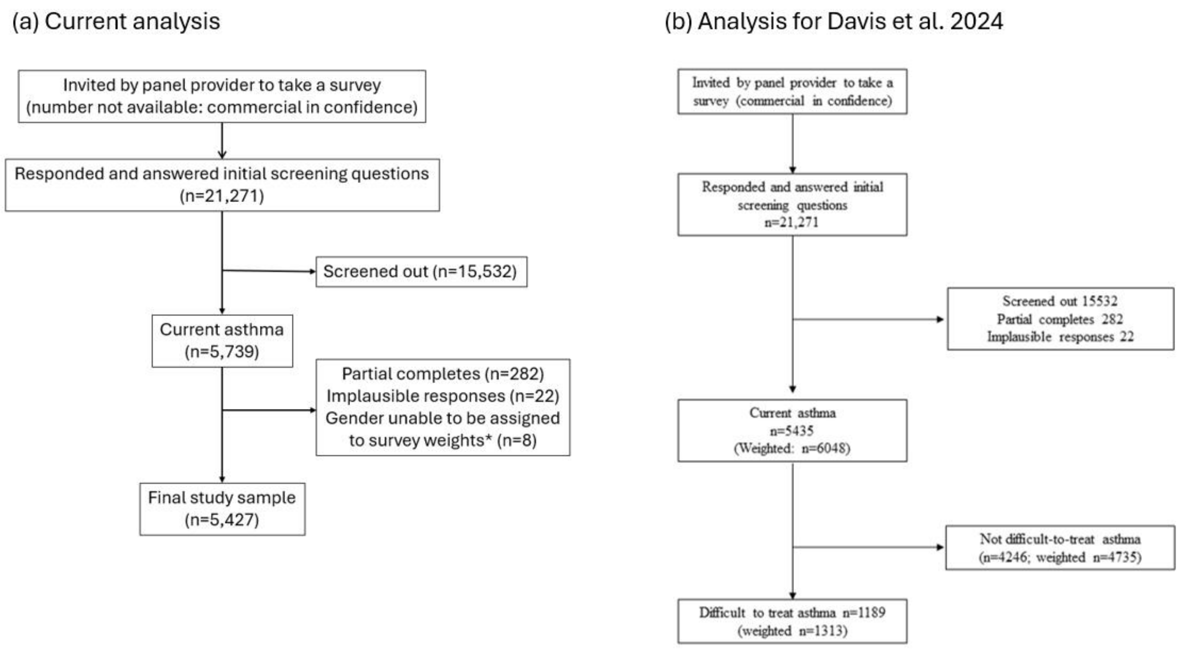

Flow chart includes all data available from the panel provider.

*8 respondents selected “other” for the question about gender, with open text responses such as “non-binary”, “gender fluid”. These respondents were unable to be included in the analysis due to the unavailability of an “other” category in the National Health Survey 2017-18 population, which was used in the weighting calculation and was not offered as an option in the 2012 survey.

The difference in numbers between (a) the current analysis and (b) the analysis of difficult-to-treat and non-difficult-to-treat asthma from the same survey reported in Davis et al. 2024 [1] is due to the differences in weighting required for the two analyses. The present analysis, comparing data from 2012 (n=2,686) and 2021 (n=5427), was weighted based on age, gender and state/territory of residence. In the Davis et al. 2024 analysis [1], which compared data for those with difficult-to-treat asthma versus non-difficult-to-treat asthma, it was also necessary to include SEIFA in the weights due to differences in SEIFA by difficult-to-treat asthma status, giving a weighted total of n= 6048.

The target sample size of 5000 was based on the prevalence of outcomes of interest and desired precision. For example, for a variable with a prevalence of 50%, the 95% confidence limits would be 48.6–51.4%, for a prevalence of 10%, they would be 9.18–10.9%, and for a prevalence of 5%, they would be 4.4–5.6%.

Table S1 – CHERRIES checklist for 2012 and 2021 surveys

		2012*	2021#
Design	Design and target population	This was a web-based survey of people aged ≥16 years with current asthma, selected randomly from amongst members of a large Australian web-based panel held by Survey Sampling International.	This was a web-based survey of people aged ≥18 years with current asthma, selected randomly from amongst members of a large Australian web-based panel held by Dynata (previously known as Survey Sampling International) and two additional population panels.
Ethics	Ethics approval	Ethics approval was obtained from the Human Research Ethics Committee at Repatriation General Hospital Concord, Sydney, Australia (approval CH62/6/2012-121; LNR/12/CRGH/153)	Ethics approval was obtained from the Human Research Ethics Committee at University of Sydney, Sydney, Australia (approval #2020-630).
	Informed consent	All participants, as members of a web-based panel, had already provided informed consent to participate in online surveys. Informed consent for the present survey was obtained from all those agreeing to complete a survey, with participants informed on the welcome page that the survey concerned health issues, that it would take approximately 25 minutes to complete, that all responses were confidential and anonymous and that reporting would be on an aggregate level only. Consent was indicated when respondents clicking the 'Go to Survey' button from this page.	All participants, as members of a web-based panel, had already provided informed consent to participate in online surveys. Informed consent for the present survey was obtained from all those agreeing to complete a survey, with participants informed on the welcome page that the survey concerned health issues, that it would take approximately 15 minutes to complete, that all responses were confidential and anonymous and that reporting would be on an aggregate level only. Consent was indicated when respondents clicked the 'Go to Survey' button from this page.
	Data protection	Proprietary survey software and local servers were used to ensure data protection. No personal information was linked to survey results in any way. The fully de-identified dataset is kept on password protected computers.	Proprietary survey software and local servers were used to ensure data protection. No personal information was linked to survey results in any way. The fully de-identified dataset was kept on password protected computers in the University Data Store.
Development and pre-testing		The survey instrument was designed using input from studies, the outcomes from earlier qualitative research and healthcare professional review. A draft survey instrument was cognitively tested with five people with asthma, and modified following cognitive debriefing and evaluation of comprehension, interpretation, memory retrieval, summarising of information and availability of appropriate responses. The survey was then pilot tested with approximately 600 respondents.	The survey instrument was designed using input from literature searches for existing questions and questionnaires where available- including outcomes from earlier qualitative research and healthcare professional review.<br>A draft of the survey instrument was cognitively tested by four people, and modified following evaluation of comprehension and interpretation. After the survey was piloted, questions about biological medication use were expanded to better define users.
Recruitment process	Open vs closed survey	This was an open survey. An online panel provider, Survey Sampling International (SSI), was engaged to use proportionate stratification sampling to recruit a cohort of people with asthma, representative of Australian populations with asthma based on age, gender and state of residence, from their panel of potential survey participants.<br>Recruitment to SSI's online panel is achieved in multiple ways, predominately through banner advertisements, invitations and messaging via relationships with other websites, online communities and social media groups. SSI undertakes active strategies with communities of interest of all types to incorporate rare populations into the online sample blend. Potential participants go through rigorous quality screening before being accepted onto a panel."	This was an open survey. The online panel provider Dynata used proportionate stratification sampling to recruit a cohort of people with current asthma, representative of Australian populations with asthma based on quotas for age-group (5), gender (2) and state/territory of residence (8) (giving a total of 80 cells) based on data from the most recent National Health Survey), from their panel of potential survey participants.<br>Recruitment to Dynata's online panel is achieved in multiple ways, predominantly through banner advertisements, invitations and messaging via relationships with other websites, online communities and social media groups. Dynata undertakes active strategies with communities of interest of all types to incorporate rare populations into the online sample blend. Potential participants go through rigorous quality screening before being accepted onto a panel.
	Contact mode	To minimise bias, participants underwent a three-stage randomisation process. Firstly, potential participants were selected at random from SSI's panel to be invited to 'take a survey'; no information about the survey topic was provided at this stage. A set of profiling questions was randomly selected or respondents to answer, one of which was the question 'Have	To minimise bias, participants underwent a three-stage randomisation process. Firstly, potential participants were selected at random from Dynata's panel to be invited to 'take a survey'; no information about the survey topic was provided at this stage. A set of profiling questions included age-group, gender and postcode of residence, together with a question 'Have you ever experienced any of the following conditions?' of which

		2012*	2021#
		you ever experienced any of the following conditions?' of which 'asthma' was one possible option. Those choosing 'asthma' were directed to the screener page of the present survey.	'asthma' was one possible option. Those choosing 'asthma' were directed to the screener page of the present survey.
	Advertising the survey	The survey was not advertised; as above, randomly-selected members of the online panel were invited to participate.	The survey was not advertised; as above, randomly-selected members of the online panel were invited to participate.
Survey administration	Web/email	This was a web-based survey, with respondents channelled through SSI's participant interface to UltraFeedback's online survey site. Responses were collected through the online survey platform and stored on secure local servers. Responses were multiple choice, numeric, and open text.	This was a web-based survey, with respondents channelled through Dynata's participant interface to the online survey site. Responses were collected through the online survey platform and stored on secure local servers. Responses were multiple choice, numeric, and open text.
	Context	The panel provider, SSI, is a research services company who maintain a pool of potential respondents for research projects.	The panel provider, Dynata, is a research services company that maintains a pool of potential respondents for research projects.
	Mandatory/ voluntary	Voluntary. Prospective respondents were screened to ensure that they had current asthma and were the appropriate age. Proportionate sampling was used in order to match the population of people in Australia with current asthma.	Voluntary. Prospective respondents were screened to ensure that they had current asthma and were the appropriate age. Proportionate sampling by age-group, gender and state/territory of residence was used in order to match the population of people in Australia with current asthma.
	Incentives	Respondents were incentivised for their participation by the panel provider, SSI, with points which can be redeemed for money. The points obtained for participation in the present survey were estimated to have a value of AUD1.50.	Respondents were incentivised for their participation by the panel provider, Dynata, with points which can be redeemed for money. The points obtained for participation in the present survey were estimated to have a value of AUD1.00-7.00 in 2021.
	Time/date	Responses were collected between November 1 to 27, 2012.	Responses were collected in February and March 2021.
	Item randomisation	No randomisation of items was used.	No randomisation of items was used.
	Adaptive questioning	Adaptive questioning (branched) was used. Relevant survey items were displayed based on previous responses (e.g. only those who had seen a GP in the previous 12 months were shown the follow-up questions about the visit(s)). Certain items were also populated based on previous responses.	Adaptive questioning (branched) was used. Relevant survey items were displayed based on previous responses (e.g. only those who had seen a GP in the previous 12 months were shown the follow-up questions about the visit(s)). Certain items were also populated based on previous responses.
	Number of items	A maximum of three items were displayed on any one survey page. The full survey comprised a total of 117 items, although because of the adaptive nature of the questionnaire, not all respondents answered all items.	The full survey comprised 40 questions; however because of the adaptive nature of the questionnaire, not all respondents answered all items.
	Number of screens	The full survey was distributed over approximately 70 screens	The full survey was distributed over approximately 77 screens.
	Completeness check	All survey items were deemed to be mandatory, and respondents prompted to complete outstanding items before leaving the survey page on which the item was contained. Most items, except screener items and those items required for adaptive questioning included a 'Don't know/ not sure' option.	All survey items were deemed to be mandatory, and respondents prompted to complete outstanding items before leaving the survey page on which the item was contained. Many items, except screening items and those items required for adaptive questioning included a 'Don't know/ not sure' option.
	Review step	Respondents were unable to change their responses once submitted. The 'save answers and continue' button on each page of the survey was preceded by the sentence "Please note that when you click the [Save answers and continue] button below, your responses will be saved to the database and you will no longer be able to change them".	A 'back' or 'continue' option was provided at the bottom of each screen. Respondents were unable to change or review their answers once the survey was submitted.
Response rates	Unique site visitor	Determination of unique visitors was handled by the panel provider SSI. SSI utilise digital fingerprinting and traps for geo-IP violations to ensure that respondents only compete the survey once	The system handled the sampling automatically; Dynata was unable to tell how many invitations were sent for this particular project.
	View rate	Not applicable; respondents were invited through an external panel.	Not applicable; respondents were invited through an external panel.
	Participation rate	80,518 panel members were randomly invited to complete a survey; 27,606 responded giving a panel participation rate of 34.29%. Of the 3,033 potential	Data about the number of panel members invited to complete a survey (before identification of those with asthma) were not available from Dynata. The authors of the

		2012*	2021#
		respondents with current asthma who were invited to participate in the survey, 15 did not proceed to the survey, giving a participation rate from the current asthma population of 99.51%	2012 advised us that they had been provided in error with additional numbers for their flow-chart; these additional data should have been commercial in confidence). The number of those who agreed to participate after quotas were applied was 5739
	Completion rate	Of the 3,018 respondents who commenced the survey, 2,686 completed it, giving a completion rate of 89.00%.	5457/5739 = 95.1%
Preventing multiple entries from same individual	Cookies used	No	Yes
	IP check	SSI utilise digital fingerprinting and traps for geo-IP violations to ensure only unique respondents completed the survey.	Yes, and digital fingerprinting was used to make sure there were unique respondents.
	Log file analysis	Not used	Not used
	Registration	Entry to the survey was via a unique login provided to each invitee to the survey.	Entry to the survey was via a unique login provided to each invitee to the survey.
Analysis	Handling of incomplete questionnaires	Only completed questionnaires were included in the final dataset.	Only completed questionnaires were included in the final dataset.
	Questionnaires with atypical timestamp	No respondents were removed from the survey for completing the items too quickly. The minimum completed survey was timed at approximately 13 minutes. No 'straight-liners' were identified in post-hoc tests	Participants who completed the survey too speedily were removed
	Statistical correction	Satisfactory numbers were achieved for all demographic sub-groups, although some quotas were applied mid-way through the data collection process in order to more accurately match the sample frame of people with asthma in Australia	Satisfactory numbers were achieved for all demographic sub-groups, with quotas applied throughout data collection to match the sample frame of people with asthma in Australia by age-group, gender and state/territory of residence. Data for comparisons between 2012 and 2021 were adjusted for Socio-Economic Indexes for Areas (SEIFA) Index of Relative Socio-economic Disadvantage quintile.

* CHERRIES checklist for 2012 survey, repeated with permission from Reddel et al, *Med J Aust* 2015, Appendix 1 [2].

\# CHERRIES checklist for 2021 survey, repeated with permission from Davis et al, *Respirology* 2024, Table S1 [1]..

Table S2: Demographics of populations included in 2012 and 2021 surveys compared with contemporaneous Australian government National Health Survey data for people with asthma

	Survey Nov 2012	National Health Survey* 2011/2012	Survey Feb 2021	National Health Survey† 2017/2018
	N=2686		N=5427	
**Age group, years**				
<20[#]	207 (7.7%)	3.8%	116 (2.1%)	3.5%
20-29	493 (18.4%)	18.8%	848 (15.6%)	17.4%
30-39	523 (19.5%)	18.2%	985 (18.2%)	19.5%
40-49	377 (14.0%)	18.3%	1056 (19.5%)	16.9%
50-59	441 (16.4%)	16.6%	848 (15.6%)	16.0%
60-69	344 (12.8%)	12.9%	979 (18.1%)	13.5%
70+	302 (11.2%)	11.5%	596 (11.0%)	13.3%
**State/territory of residence**				
NSW	836 (31.1%)	32.5%	1637 (30.2%)	32.3%
ACT	38 (1.4%)	1.7%	101 (1.9%)	1.7%
Vic	709 (26.4%)	25.3%	1484 (27.3%)	26.2%
Tas	61 (2.3%)	2.3%	140 (2.6%)	2.2%
Qld	528 (19.6%)	19.9%	1174 (21.6%)	19.7%
SA	214 (8.0%)	7.4%	383 (7.1%)	7.1%
WA	292 (10.9%)	10.3%	481 (8.9%)	10.2%
NT	9 (0.3%)	0.8%	28 (0.5%)	0.7%
**Remoteness area‡**				
Major cities of Australia	1947 (72.9%)	71.3%	3550 (65.6%)	72.8%
Inner regional Australia	505 (18.9%)	18.9%	1121 (20.7%)	17.8%
Other	220 (8.2%)	9.8%	742 (13.7%)	9.3%
**SEIFA Index of Relative Socio-economic Disadvantage**				
Most disadvantaged	315 (11.8%)	17.8%	813 (15.0%)	17.6%
SES 2	381 (14.3%)	20.5%	998 (18.4%)	20.5%
SES 3	557 (20.9%)	20.9%	1113 (20.6%)	20.6%
SES 4	681 (25.5%)	20.1%	1097 (20.3%)	20.7%
Least disadvantaged	734 (27.5%)	20.7%	1391 (25.7%)	20.6%
Employment status				
Employed	1468 (54.7%)	67.0%	3429 (63.2%)	65.5%
Unemployed	199 (7.4%)	2.7%	420 (7.7%)	2.9%
Not in the labour force	1018 (37.9%)	30.4%	1578 (29.1%)	31.6%

Survey weights were applied to the 2012 and 2021 surveys based on National Health Survey data for prevalence of current asthma by age group, gender and state/territory of residence.

* Australian Bureau of Statistics (2011-12). Microdata: Australian Health Survey, National Health Survey, 2011-12 (Microdata Download), accessed 15 November 2024.

† Australian Bureau of Statistics (2017-18). Microdata: National Health Survey, 2017-18 (Microdata Download), accessed 15 November 2024.

#16–19 years for the 2012 web-based survey population and the NHS populations; 18–19 years for the 2021 web-based survey.

‡ Remoteness area based on Australian Statistical Geography Standard (ASGS) by postcode of residence.

Table S3 (data for Figure 1): Health care utilisation for asthma in the previous 12 months, 2012 and 2021

Type of health care utilisation, n (%)	2012	2021	aOR*	95% CI	*P*-value[#]
Number of participants	N=2686	N=5427			
Urgent health care for asthma in past 12 months					
Urgent GP visit for asthma	627 (23.3)	1902 (35.0)	1.78	1.59 – 1.98	<0.001
Hospital or emergency department visit for asthma	269 (10.0)	1316 (24.3)	2.96	2.55 – 3.44	<0.001
Spent at least one night in hospital for asthma	98 (3.7)	920 (16.9)	5.38	4.31 – 6.72	<0.001
Any of the above	769 (28.6)	2056 (37.9)	1.53	1.37 – 1.69	<0.001
Non-urgent GP visit for review of asthma in past 12 months	1355 (50.4)	3588 (66.1)	1.87	1.70 – 2.06	<0.001

aOR: adjusted odds ratio; CI: confidence interval; GP: general practitioner.

*Data were adjusted for age group, gender and Socio-Economic Indices for Area (SEIFA) Index of Relative Socio-economic Disadvantage quintile.

[#]Within each group, the odds of having healthcare utilisation between 2012 and 2021 were analysed by a logistic regression model, adjusted for age group, gender and SEIFA.

Table S4: Reasons for poor adherence with ICS-containing medications

Participants reporting any use of ICS-containing medication during the previous 4 weeks (n=2,515) were asked: "*During the last 4 weeks, have any of the following prevented you from taking any [name of medication] as prescribed?*"

Factor	N (%)
Feeling well at the moment	631 (25.1%)
I don't think I need it	328 (13.1%)
The cost of the medication	266 (10.6%)
Lack of time	177 (7.0%)
Concern about potential side-effects	176 (7.0%)
Concern about side-effects that I have experienced	136 (5.4%)
Concern about interactions with other medications	108 (4.3%)
Lost my medication	109 (4.3%)
Forgot my medication	359 (14.3%)
Difficulty using the device	73 (2.9%)
Ran out of the medication	165 (6.6%)
None of the above	1288 (51.2%)

Note: Multiple responses were permitted.

Table S5: Asthma symptom control and frequency of ICS-containing preventer use in (A) 2012 and (B) 2021

Table S5A. Asthma symptom control and frequency of ICS-containing preventer use over the past 12 months in 2012 (n = 2654*)

				Asthma symptom control (ACT score)						
	Total			20-25		16-19		5-15		
**Use of ICS**	n	%		n	%	n	%	n	%	
Every day	845	31.8%	***B 388 (14.6%)***	368	25.4%	225	37.4%	252	41.8%	***C 522 (19.7%)***
5 or 6 days a week	65	2.4%		20	1.4%	22	3.7%	23	3.8%	
3 or 4 days a week	91	3.4%	***A 1063 (40.0%)***	34	2.3%	33	5.5%	24	4.0%	***D 682 (25.7)***
1 or 2 days a week	111	4.2%		43	3.0%	30	5.0%	38	6.3%	
Less than one day a week	108	4.1%		56	3.9%	31	5.2%	21	3.5%	
Less than one day a month	94	3.5%		66	4.5%	17	2.8%	11	1.8%	
Only when I exercise**	18	0.7%		12	0.8%	6	1.0%	0	0.0%	
A few times a year **	269	10.1%		207	14.3%	45	7.5%	17	2.8%	
Did not use ICS	1054	39.7%		645	44.4%	192	31.9%	217	36.0%	
All	2655	99.9%		1451	100.0%	601	100.0%	603	100.0%	

Table S5A has been reproduced with permission from Reddel et al, *Med J Aust* 2015; 202: 492-497 [2].
ICS = inhaled corticosteroids
Groups A to D correspond to the categories shown in Figure 2: A = well controlled symptoms with infrequent or no preventer use; B = well controlled symptoms with regular preventer use; C = poorly controlled symptoms with frequent preventer use; D = poorly controlled symptoms with infrequent or no preventer use.
Disparity between the sum of the numbers and the total number of participants is because of rounding weighted data.
*32 participants who had used both an ICS-only and a non-ICS preventer in the past 12 months were excluded from this analysis because the questionnaire structure did not permit the frequency of use of the ICS to be distinguished.
**Questions asked only in 2012.

Table S5B. Asthma symptom control and frequency of ICS-containing preventer use in 2021 (n = 5427)

				Asthma symptom control (ACT score)						
	Total†			20-25		16-19		5-15		
**Use of ICS**	n	%		n	%	n	%	n	%	
Every day	1353	24.9%	***B†† 797 (14.7%)***	707	27.1%	313	22.9%	333	22.9%	***C†† 758 (14.0%)***
5 or 6 days a week	202	3.7%		90	3.5%	60	4.4%	52	3.5%	
3 or 4 days a week	374	6.9%	***A†† 1807 (33.3%)***	98	3.7%	113	8.2%	164	11.3%	***D†† 2066 (38.1%)***
1 or 2 days a week	370	6.8%		135	5.2%	106	7.7%	130	8.9%	
Less than one day a week	216	4.0%		165	6.3%	33	2.4%	19	1.3%	
Less than one day a month#	790	14.6%		339	13.0%	212	15.5%	238	16.4%	
Not used in last 12 months	2122	39.1%		1070	41.1%	531	38.9%	520	35.7%	
All	5427	100.0%		2604	100.0%	1367	100.0%	1456	100.0%	

ICS = inhaled corticosteroids
Groups A to D correspond to the categories shown in Figure 2: A = well controlled symptoms with infrequent or no preventer use; B = well controlled symptoms with regular preventer use; C = poorly controlled symptoms with frequent preventer use; D = poorly controlled symptoms with infrequent or no preventer use.
Disparity between the sum of the numbers and the total number of participants is because of rounding weighted data.
#Used in last 12 months, but not in last 4 weeks.
†P < 0.001 (Comparison of frequency of ICS-containing preventer use between 2012 and 2021: Cochran-Armitage test for trend).
††P < 0.001 (Comparison of frequency of ABCD groups between 2012 and 2021: Cochran-Armitage test for trend).

Table S5C: Proportion of participants requiring urgent healthcare in the previous 12 months, by symptom control/adherence group in Figure 2 (b)

Group	Description	Urgent healthcare, n (%)		
		**2012**	**2021**	**P-value**
A	Good symptom control with no ICS-containing medication or poor adherence (<5 days/week)	158/1063 (14.9%)	273/1807 (15.1%)	0.155
B	Good symptom control with good self-reported ICS adherence (≥5 days/week)	81/388 (21.0%)	108/797 (13.6%)	0.031
C	Uncontrolled symptoms despite good self-reported ICS adherence	233/522 (44.6%)	357/758 (47.1%)	0.671
D	Uncontrolled symptoms with no ICS or poor adherence	281/681 (41.2%)	1318/2066 (63.8%)	<0.001
All		753/2654 (28.4%)	2056/5427 (37.9%)	<0.001

Urgent health care = urgent GP visit or emergency department or hospital visit or spent at least one night in hospital for asthma.
Groups A to D correspond to the categories shown in Box 4.
Disparity between the sum of the numbers and the total number of participants is because of rounding weighted data.
Within each group, the difference in risk of urgent health care between 2012 and 2021 was analysed by a logistic regression model, adjusted for age-group, gender and Socio-Economic Indices for Area (SEIFA) Index of Relative Socio-economic Disadvantage.

References:

1. Davis SR, Ampon RD, Poulos LM et al. Prevalence and burden of difficult-to-treat and severe asthma in Australia: A national population survey. Respirology. 2024;29(8):685–693. https://doi.org/10.1111/resp.14722

2. Reddel HK, Sawyer SM, Everett PW, Flood PV and Peters MJ. Asthma control in Australia: a cross-sectional web-based survey in a nationally representative population. Med J Aust. 2015; 202(9): 492-7.
